# The Association Between Cytomegalovirus Infection and Kidney Damage in the Liver Transplant Setting

**DOI:** 10.3390/v16121830

**Published:** 2024-11-26

**Authors:** Ramin Raul Ossami Saidy, Franziska Eurich, Brigitta Globke, Wenzel Schöning, Robert Öllinger, Nathanael Raschzok, Johann Pratschke, Dennis Eurich, Luca Dittrich, Eva Maria Dobrindt

**Affiliations:** 1 Department of Surgery, Campus Virchow Klinikum and Campus Charité Mitte, Charité–Universitätsmedizin Berlin, Augustenburger Platz 1, 13353 Berlin, Germany; franziska.eurich@alumni.charite.de (F.E.); brigitta.globke@charite.de (B.G.); wenzel.schoening@charite.de (W.S.); robert.oellinger@charite.de (R.Ö.); nathanael.raschzok@charite.de (N.R.); johann.pratschke@charite.de (J.P.); dennis.eurich@charite.de (D.E.); luca.dittrich@charite.de (L.D.); eva-maria.dobrindt@charite.de (E.M.D.); 2 Berlin Institute of Health at Charité—Universitätsmedizin Berlin, BIH Academy, Clinician Scientist Program, 10117 Berlin, Germany

**Keywords:** liver transplantation, cytomegalovirus, chronic kidney disease, immunosuppression

## Abstract

Introduction: The development of chronic kidney disease (CKD) is a common and significant complication, contributing to morbidity after liver transplantation (LT). Cytomegalovirus (CMV) infection is common in the overall population, and relevant reinfection after LT may occur. CMV-associated kidney damage has been discussed, but the clinical significance on CKD development after LT remains unclear. Methods: A total of 745 patients who underwent LT between 2006 and 2017 were included in this retrospective analysis. Clinical data, as well as laboratory parameters, were analyzed. Univariate and multivariate analysis were performed. Results: The univariate analysis revealed significantly impaired estimated glomerular filtration rates (eGFRs) in patients with histories of CMV infection (81.4 (8–137) mL/min vs. 90.0 (5–147) mL/min; *p* = 0.004). This effect was confirmed in the multivariate analysis. Post-LT, eGFR was impaired in patients with CMV (re)infection at 6, 12, 36, and 60 months, 10 years, and 15 years after LT. Immunosuppressive levels were comparable between groups. Overall survival was negatively affected by CMV infection (*p* = 0.001). Discussion: A clinically significant detrimental impact of CMV infection on renal function was observed, that could individualize clinical risk evaluation prior and after LT further. However, the pathophysiological mechanisms behind this observation are not yet understood.

## 1. Introduction

Liver transplantation (LT) remains the only curative therapeutic option for various end-stage liver diseases (ESLDs) [1,2].

Post LT, lifelong immunosuppression (IS) remains standard therapy to prevent rejection episodes. IS itself is associated with short- and long-term complications such as chronic kidney disease (CKD), metabolic diseases, cardiovascular events, opportunistic infections, and malignancies [3,4,5,6,7,8,9,10].

Renal dysfunction and the diagnosis of CKD prior to LT have been established as independent risk factors for poorer outcomes after LT [11]. Via the hepatorenal pathway, kidney function can be impaired by the underlying liver disease and is therefore part of scoring systems, such as the Model for End-stage Liver Disease (MELD) [12,13]. Thus, the presence of CKD after LT is common and multifaceted, caused by liver disease itself and aggravated by, e.g., immunosuppression and decreasing renal function due to age [14,15]. Importantly, CKD has been linked to increased morbidity and decreased survival in patients [16,17,18].

CMV infection, as well as reactivation, after LT is one of the most relevant infections and it contributes to increases in morbidity and mortality [10,19]. CMV serostatus mismatch of LT donors and recipients has been established as a risk factor for reoccurring infection, and recommendations on the administration of pharmaceutical prophylaxis are based upon these laboratory findings [20,21,22]. CMV infection in healthy, immunocompetent individuals commonly resolves with only minor symptoms, but critically ill, immunocompromised patients are at heightened risk for severe courses [23,24,25]. The range of manifestations of CMV infections is wide, with uncharacteristic symptoms such as fever in addition to colitis, encephalitis, pneumonitis, and nephritis with proteinuria [24,26].

Impairment of graft function in kidney transplant (KT) patients with CMV infection is generally recognized, but its mechanism is not fully understood and ranges from induction of rejection to endothelial alterations [27]. Interestingly, CMV infection has been linked to increase arterial stiffness in CKD patients [28]. The extent to which glomerulopathy is caused by CMV infection in non-KT patients remains disputed, and its clinical significance is unknown [29,30,31,32,33].

To our knowledge, the significance of CMV infection in the development of CKD in LT patients has not been thoroughly studied. This work aims to explore the potential link between CMV infection and renal dysfunction in LT patients and to contextualize these findings within long-term outcomes following LT.

## 2. Materials and Methods

All adult patients undergoing LT at a single transplant center between 2006 and 2017 were considered for this retrospective study. Follow-up for this study was ended in 12/2023. Patients with multiorgan transplantation, re-transplantation, and missing data were excluded.

Clinical and laboratory parameters, such as MELD score, CMV serostatus of donor and recipient, dosage and trough levels of IS, estimated glomerular filtration rate (eGFR) prior to LT, and clinical course after LT, were extracted from our prospectively maintained database. Life-long follow-up after LT was ensured via our outpatient clinic according to a standardized schedule ranging from twice a week to every three months, depending on time after LT. Renal function was assessed preoperatively within one month prior to LT and 6, 12, 36, 60, 120, and 180 months after LT. Classification of CKD was conducted using the Kidney Disease: Improving Global Outcomes (KDIGO) guidelines for eGFRs [34]. To further analyze the course of renal function, loss of eGFR was calculated by subtracting the means of the eGFRs.

To analyze comorbidities relevant to the development of CKD, the presence of “cardiovascular disease” was stated when patients were diagnosed with arterial hypertension on medication or atherosclerosis (e.g., coronary sclerosis, peripheral arterial disease). Similarly, “diabetes” was acknowledged if patients received antidiabetic drug treatment. Age at time of LT was dichotomized into ≤60 and >60 years.

CMV infection after LT was diagnosed by positive CMV-DNA-PCR, and was classified as follows: (i) CMV viremia in asymptomatic patients; (ii) CMV syndrome in patients with typical signs and symptoms of the disease; (iii) histologically proven tissue-invasive CMV infection.

CMV prophylaxis consisted of acyclovir or valganciclovir (VGCV) and was administered as part of standard of care according to donor/recipient CMV-IgG constellation up to 6 months after LT [35]. Routinely, preemptive therapy was conducted in new-onset viremia, but individual factors were acknowledged.

IS was administered in an individualized manner due to patients’ risk profiles and comorbidities. Standard regimen consisted of calcineurine inhibitors (CNI; tacrolimus, cyclosporin A) with the addition of, e.g., mycophenolate mofetil (MMF), mammalian target of rapamycin inhibitors (mTORI), or glucocorticoids (GCs). GCs were routinely administered after transplantation and tapered until 12 weeks after LT. We focused on dosage and trough levels of tacrolimus and these values were analyzed within 2 weeks and 3, 6, 12, 36, 60, and 120 months after LT. Cumulative dosage was calculated by using the area under the curve (AUC). The impact of cumulative dosage on renal function was calculated using the 50th percentile for each time point and, accordingly, patients were subsumed into a “low exposure” group and a “high exposure” group. 

For descriptive analysis, absolute numbers (*n*) with percentages (%) were given with mean and standard deviation (SD) for normally distributed variables, or they were given as median with minimum and maximum for non-normally distributed variables. Cross tables were used in nominal variables and a *t*-test was used for normally distributed continuous variables. In cases of non-normally distributed values, a Mann–Whitney U-test or a Kruskal–Wallis test were performed. Pearson correlation was applied for metric variables. ANOVA was applied for the comparative analysis of multiple groups. For multivariate analysis, multinominal logistic regression was used to evaluate effect strength, and regression coefficient (β) and confidence interval (CI) were calculated. A two-sided *p*-value of <0.05 was considered significant. Statistical analysis was performed using SPSS Statistics Version 26.0 (IBM Co., Armonk, New York, NY, USA). Figures were created using SPSS and Microsoft Excel 2016. This study was approved by the ethics committee of Charité Universitätsmedizin, Berlin (protocol code EA1/255/20; date of approval: 20 October 2020).

## 3. Results

### 3.1. Cohort Characteristics

Overall, 745 patients undergoing LT between 2006 and 2017 were included in this study. The majority, 494 patients (66.3%), were male and the median age at transplantation was 56.3 (19.3–74.2) years. Median eGFR prior to LT was 84.80 (5–150) mL/min.

IS after LT was mainly CNI-based (*n* = 725/97.3%). At the end of follow-up, 313 (42.0%) patients were deceased and median survival after LT was 121.0 (6–213) months. For details on the patient cohort, see Table 1.

### 3.2. CMV Infection and Renal Function Prior to LT

Patients with positive CMV-IgG serostatus showed significantly lower median eGFR than patients without prior CMV infection (*p* = 0.004). Patients aged ≤60 had higher median eGFR compared to patients > 60 years (*p* = 0.011). Female patients showed significantly lower median eGFR (*p* < 0.001). Presence of cardiovascular disease at LT was also significantly associated with lower median eGFR (*p* < 0.001). Furthermore, the underlying disease leading to LT was associated with significant differences in median eGFR. MELD score showed statistically significant inverse correlation with eGFR (r = −0.323, *p* < 0.001). For details of variables, see Table 2.

Analysis of influence on kidney function prior to LT according to CKD stage showed similar results. However, distribution of renal function according to CKD classification did not significantly differ with regard to prior CMV infection (*p* = 0.059); for details, see Table 2.

Multivariate analysis confirmed significantly higher eGFR in patients < 60 years (*p* < 0.001) at LT and male patients (*p* < 0.001). EGFR was significantly lower in patients diagnosed with cardiovascular disease (*p* = 0.004) and higher MELD score (*p* < 0.001) in this analysis. CMV infection prior to LT did not show any statistically significant association with eGFR levels (see Table 3).

### 3.3. CMV Infection and Renal Function Post LT

EGFR after LT showed a slight increase within 6 months from a median of 84.80 (5–150) mL/min prior to LT to 86.2 (8–139) mL/min (*p* > 0.001) and a continuous decrease in the following years to 61.0 (5–118) mL/min after ten years. Statistically significant inverse correlation of MELD score and eGFR was found 6 and 12 months after LT (*p* = 0.008 and *p* = 0.006, respectively) but not at a later timepoint. Using the CKD classification, significantly higher MELD scores were observed in patients with declining kidney function 6 and 12 months (*p* = 0.002 and *p* = 0.002) after LT but not at a later point in time. 

Statistically significant decreased eGFR was observed in patients with post-LT CMV infection 6 months (*p* = 0.002), 12 months (*p* = 0.024), 36 months (*p* = 0.003), 60 months (*p* = 0.001), 10 years (*p* = 0.037), and 15 years (*p* = 0.032) after LT. Of note, comparison of these groups showed significantly higher eGFR prior to LT in patients without post-LT CMV infections (87.5 (5–150) vs. 79.9 (10–137) mL/min, *p* = 0.007). Total difference in median eGFR was 3.75 mL/min 6 months after LT and increased to 6.9 mL/min after 5 years and to 6 and 32 mL/min after 10 and 15 years, respectively.

Using the CKD classification, similar results were found: patients with post-LT CMV infection showed significantly higher stages at 6 months (CKD stage > 2: 80 (17.3%) vs. 68 (26.0%), *p* = 0.006), 3 years (CKD stage > 2: 75 (19.6%) vs. 60 (29.3%), *p* = 0.05), and 5 years (CKD stage > 2: 72 (22.6%) vs. 61 (36.5%), *p* = 0.008) after transplantation.

No difference between these two groups were found regarding age, sex, cardiovascular disease, or diabetes. Indication leading to LT showed fewer alcoholic ESLD (41.4% vs. 39.9%) and viral hepatitis (25.9% vs. 16.6%) patients developing post-LT CMV infection than patients with autoimmune diseases (12.2% vs. 14.4%) and cryptogenic cirrhosis (5.9% vs. 11.1%) (*p* = 0.003). Patients who developed a post-LT CMV infection had a higher median MELD score (14 (6–44)) vs. 18 (6–48), *p* < 0.001).

No difference was found in regard to the extent of infection (viremia vs. syndrome vs. tissue invasion) and kidney function at any time point, neither through analyzing eGFR nor CKD stages. The extent of viremia was not associated with changes in eGFR or CKD stage at any time point. A subgroup analysis of 47 patients with more than one episode of new viremia after LT vs. 224 patients with only one episode of post-LT CMV infection showed that statistically lower eGFRs were only present ten years after transplant. Here, multiple recurrent infections were associated with lower eGFR (58.0 (16–118) vs. 40.0 (8–95) mL/min, *p* = 0.018). All patients with more than one episode of post-LT CMV infection were deceased 15 years after LT.

**Figure 1:**
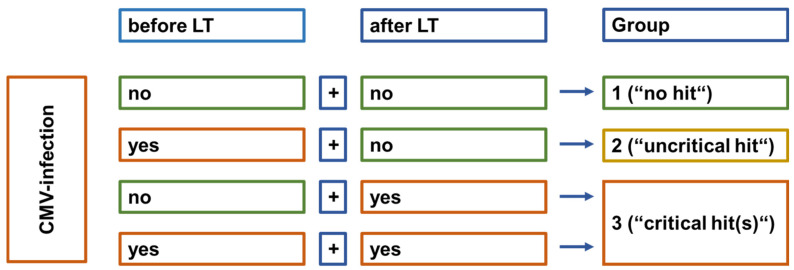
Constitution of groups for analysis of impact of CMV infection. For the analysis of subgroups in regard to exposition to CMV, three groups were defined. Group 1 is formed by patients without any recorded CMV infection; in group 2, patients were only CMV-IgG-positive at LT; group 3 was formed by patients that had recorded CMV infection after LT.

For further analysis, three groups were defined: group 1—patients with CMV-negative serostatus at the time of LT and no recorded post-LT CMV infection; group 2—patients with seropositive CMV status at the time of LT without post-LT CMV infection; group 3—all patients with post-LT CMV infection regardless of the preoperative serostatus (see Figure 1).

No difference was observed between groups regarding age, sex, or comorbidities, but MELD score was different with 13 (6–40) in group 1, 15 (6–40) in group 2, and 18 (6–40) in group 3 (*p* < 0.001).

eGFR between these groups differed prior to LT with statistical significance, as group 1 showed better median eGFR with 90.0 (5–150) mL/min than group 2, with a median eGFR of 84.3 (8–137) mL/min, and group 3 with 79.9 (10–137) mL/min (*p* = 0.005). This observation remained statistically significant throughout the follow-up of 6 months, 3 years, and 5 years (*p* = 0.005, *p* = 0.011, and *p* = 0.005, respectively). In the long-term follow-up, however, 10 and 15 years after LT, this effect did not reach statistical significance anymore (*p* = 0.11 and *p* = 0.11, respectively).

**Figure 2:**
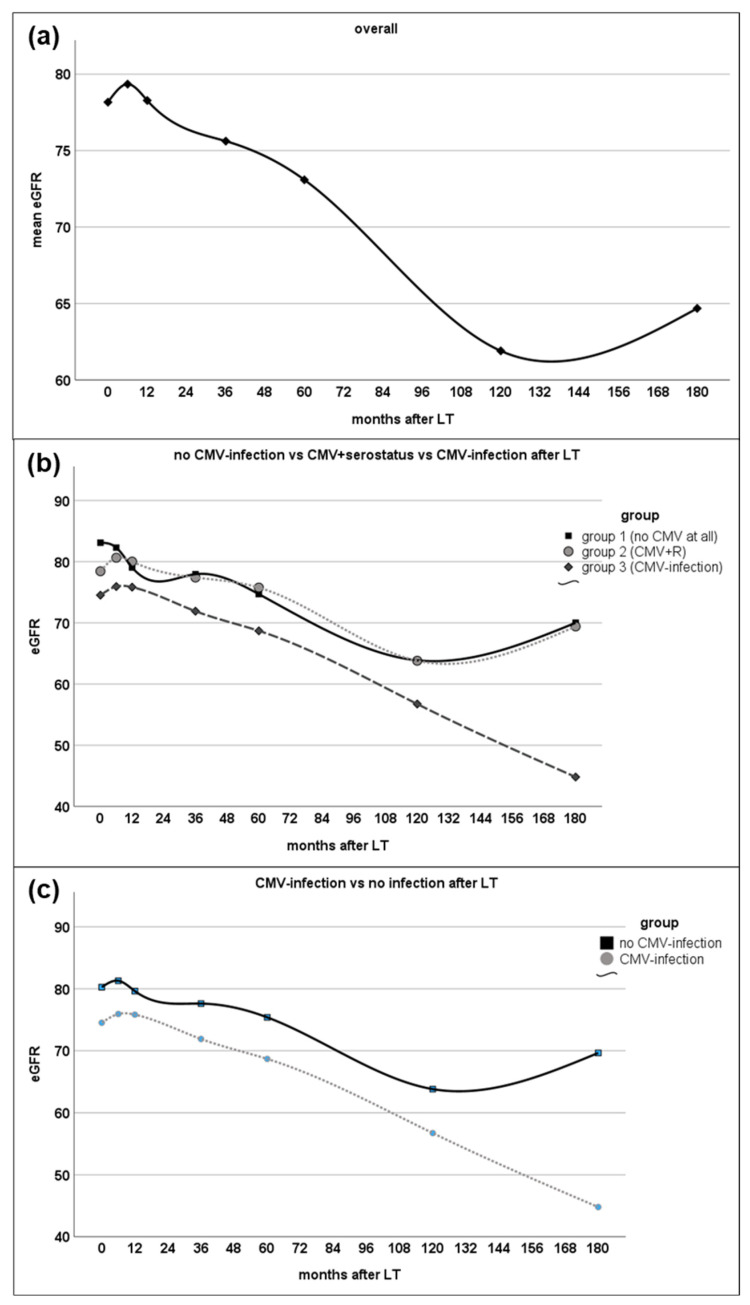
Course of median eGFR over time. There was a decrease in overall renal function regarding median eGFR over time in the overall cohort (**a**). eGFR showed different dynamics depending on occurrence of CMV infection (**b**,**c**). For details on eGFR at given time points, see Supplementary Materials, Table S1. LT—liver transplantation; CMV—cytomegalovirus; ~ indicates interpolation line.

Analyzing renal function using the CKD classification showed no statistically significant differences in the distribution of the CKD stages between the groups at the time of transplant (*p* = 0.071). Six months after LT, CKD stages ≥ 3 were found more frequently in group 3 (25.9%) vs. group 2 (17.5%) or group 1 (17.0%) (*p* = 0.015). This statistically significant difference in CKD stages was not found at the timepoints of 1 and 3 years, but was found again at 5 years (*p* = 0.038) after LT. For details on the course of eGFR, see Figure 2 and Supplementary Materials, Table S1.

### 3.4. Immunosuppression and Renal Function

To assess the impact of immunosuppression on kidney damage, the dosage of CNI was compared between groups. Mean tacrolimus trough level as well as cumulative dosage showed no difference between patients with or without CMV infection after LT at any time point. Cross-testing of dichotomized cumulative dosage was conducted, using the 50th percentile as the cut-off; here, statistically significantly lower dosages were found more frequently in the patients with CMV infection at 6 months after LT (*p* = 0.009) but not beyond.

Comparison of patients with no CMV infection (group 1), patients with CMV seropositivity only (group 2), and those with CMV infection (group 3) also showed no significant difference in mean tacrolimus trough level or cumulative exposure to CNI. Here, dichotomized tacrolimus exposure again revealed the lowest cumulative tacrolimus dosage in group 3 with statistical significance only at 6 months and 12 months after LT (*p* = 0.015, *p* = 0.014).

Analysis of “low” or “high” tacrolimus exposure and CKD classification showed significant differences between these groups only 12 months after LT: here, the group of “low exposure” consisted of more patients with impaired renal function (CKD > 1, *p* = 0.011). No difference in eGFR was found at any time point after LT between patients undergoing rejection prophylaxis with monotherapy or combination therapy. Analysis of IS regimen (monotherapy vs. combination therapy) showed no significant differences on CMV occurrence after LT. Usage of mTORI in IS regimen revealed a trend towards decreased CMV infections (*n* = 25 (28.7%) vs. *n* = 246 (37.6%), *p* = 0.067).

### 3.5. CMV Infection and Overall Survival

**Figure 3:**
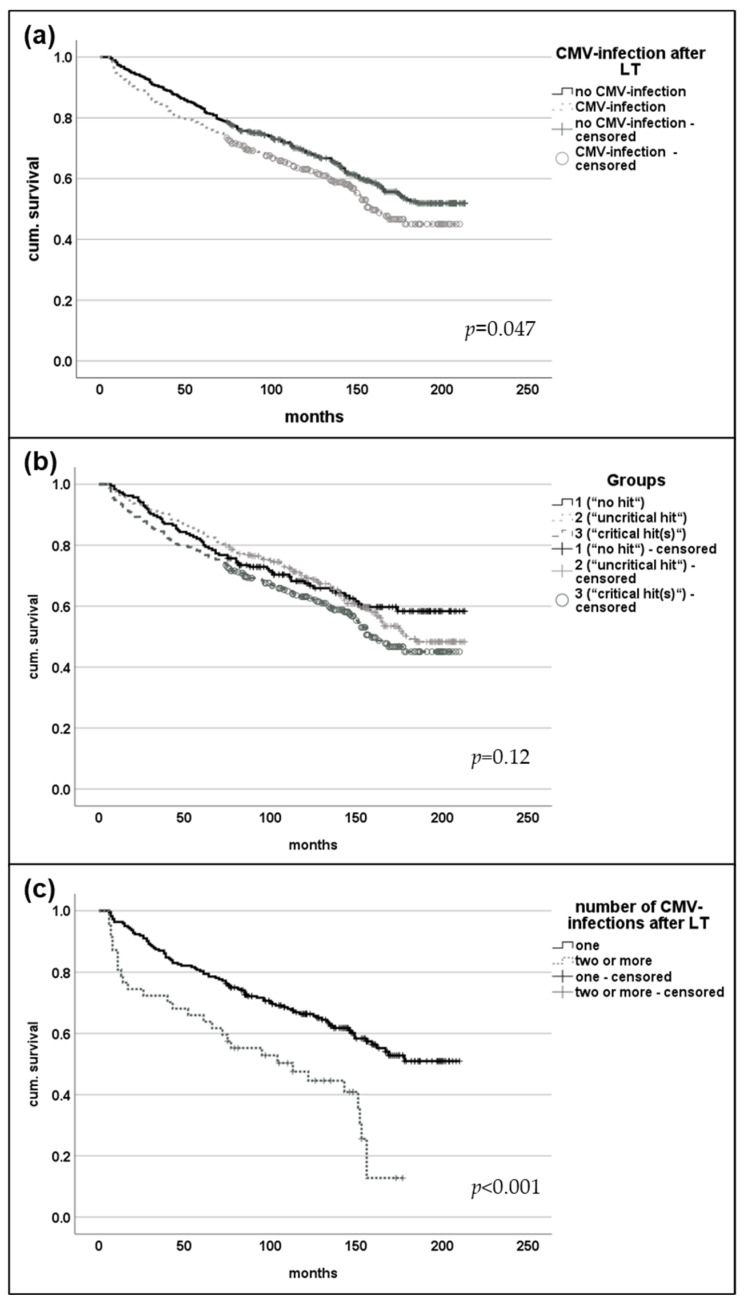
Survival analysis after LT. Kaplan–Meier analysis revealed significantly impaired overall survival after LT in patients with recorded CMV infection after transplantation (**a**). Subgroup analysis for patients without any recorded CMV exposure (Group 1) compared to patients with CMV IgG positivity prior to LT (group 2) or patients with CMV infection after LT (group 3) did not show statistically significant differences (**b**). Patients with more than one episode of CMV viremia were found to have impaired overall survival after LT (**c**).

Median survival after LT was significantly longer in seropositive CMV (CMV + R) patients prior to LT compared to seronegative recipients (128.0 (6–213) vs. 110.5 (6–113) months, *p* = 0.048). However, CMV infection after LT was associated with an impaired overall survival of 114.0 (0–210) compared to 126.5 (6–213) months (*p* = 0.001). This observation was confirmed using a Kaplan–Meier analysis (Log rank 0.047). A similar difference was found between the three groups. Patients with a history of CMV infection prior to LT (group 2) had the highest median survival of 129.0 (6–213) months, followed by patients without any CMV infection at all with 117.0 (7–213) months. Patients with CMV infection after LT (group 3) showed shortest median survival of 114.0 (6–210) months (*p* = 0.003). In Kaplan–Meier analysis, this observation did not reach statistical significance (*p* = 0.12). Patients with more than one CMV infection after LT also showed decreased median survival (81.0 (6–177) vs. 120.5 (6–210) months, *p* = 0.001), and this effect was confirmed in the Kaplan–Meier analysis (log-rank < 0.001) (see also, Figure 3). Malignancies were the most common cause of death in groups 1 (58.2%) and 2 (45.9%), whereas infections were the most common cause of death in group 3 (34.3%, *p* = 0.029). MELD score did not correlate with overall survival (*p* = 0.815).

## 4. Discussion

In this study, reporting on 745 LT patients from a single center, a significant impact of CMV infection after LT and reduced kidney function was found up to 15 years after transplantation.

Reduced glomerular filtration rate was observed in patients with a history of CMV infection prior to LT, as defined by IgG positivity in univariate but not multivariate analysis. Other potentially confounding factors (age, sex, indication for LT) also showed significant association to reduced kidney function, but diagnosis of diabetes at the time of transplantation did not. However, we did not assess the severity of diabetes (e.g., insulin dependency) or new-onset diabetes during follow-up. Of note, we found a strong correlation of MELD score and renal function up to 12 months after LT but not on overall survival. Correspondingly, higher MELD scores have been associated with more frequent acute kidney injury and even increased short-term mortality after LT [36,37,38].

In this study, CMV-IgG-positive patients showed lower eGFR prior to LT compared to those without. Prevalence of CMV worldwide is high, ranging between 50 and 100%, and infection is usually successfully controlled by the immune system in the immunocompetent population [39,40]. Of note, CMV serology cannot predict immunity [41]. Furthermore, reduced renal function in patients with ESLD is common and associated with impaired outcome [11]. While only few reports of CMV-related kidney damage in immunocompetent patients exist, a link to glomerulopathy and arterial stiffness has been made [28,29,31]. Also, CMV copies in the urine sediment in non-immunocompromised patients with acute kidney injury have been reported [42]. While renal damage in pre-LT patients is certainly multifaceted, it is noteworthy that ESLD patients can be classified as immunocompromised with dysfunction in both innate and acquired immunity [43,44,45]. CMV seropositive cirrhotic patients have been reported to have an elevated mortality [46]. The impact of CMV infection before and after LT on the development of CKD in LT patients is not very well known. In this study, significantly reduced eGFR was found in patients with CMV infection after LT and in the group of patients with recurrent CMV exposition compared to CMV-negative patients. However, these groups showed differences in renal function even prior to LT. This might be due to the fact that there is a well-known significant overlap of CMV seropositive patients prior to LT and infection after LT, as CMV seropositivity contributes to a higher rate of reinfection after transplantation [47,48,49]. International recommendations for prophylactic treatment regimens take these risk constellations into account [20,48,50,51]. No association between amounts of episodes of CMV viremia after LT or extent of CMV infection and kidney damage was observed in this cohort. Given the relatively low numbers of patients in the group of CMV syndrome (*n* = 66) and tissue invasion (*n* = 2), the effect of extent of CMV infection might have been missed in statistical analysis. Additionally, the CMV-induced renal damage might not be connected to the typical clinical presentation of the infection.

The mechanism and dynamics of the hypothesized CMV-induced damage on glomeruli remain unclear and were not investigated in this work. However, results from this study and clinical data would rather suggest a “slow-burning” processes linked to pathophysiology with subclinical manifestation rather than fulminant kidney injury. This is supported by findings of persistent elevated mortality in patients with CMV infection after liver or kidney transplantation in the long-term setting, despite the early occurrence of infection post-transplantation [52,53,54]. Direct glomerulopathy as well as, e.g., affection of vessels, has been proposed to contribute to kidney damage in CMV infection [55,56,57,58]. Others have evaluated CMV-induced glomerulopathy rather as episodes or manifestation of rejection that have been misidentified in the context of KT patients [59]. As mentioned above, in LT patients, negative effects of CMV infection are widely known, but the affection of the kidney is not the focus [47,60]. Assessing different “groups at risk” showed clinically relevant impaired kidney function in patients with histories of CMV infection prior to LT and CMV infection after LT, even when compared to those with a history of CMV infection prior to LT only. This might suggest additional, cumulative damage caused by CMV infection, as relevant confounders such as age and sex were ruled out. Certainly, multiple factors contribute to renal damage in an additive maybe even exponential character and especially in multimorbid patients with ESLD or after LT. Weighting of factors is difficult, but CMV infection might mean an additional “hit” in a multifactorial process.

CNIs, especially tacrolimus, were the backbone IS in this cohort and are known for their dosage-dependent nephrotoxicity [61]. However, this potentially major confounder was not present in this study, as mean levels and cumulative dosages were not higher in patients with impaired renal function. On the contrary, significantly lower CNI exposure was observed 6 months after LT, indicating proper trough-level-dependent monitoring and individualized adjustment of IS.

In summary, these findings highlight the importance of prevention of CMV reactivation in the post-transplant setting and underline the effect of individualized regimens in terms of prevention strategies, closely linked to a well-observed individualized immunosuppressive therapy.

Certain limitations must be mentioned. Although the cohorts mostly represent current standards of diagnostics and treatment, its retrospective nature might have inherited relevant additional confounders in diagnostics and therapeutical concepts that were not exposed and therefore not taken into account in statistical analysis. Renal function prior to LT is certainly affected by various aspects including comorbidities and, e.g., socioeconomic aspects, that were not all assessed in this study. Also, the fundamental question of the pathophysiological mechanisms of CMV-induced renal damage was not explored in this study; thus, at this point, only its clinical observation remains. However, as it seems to be of relevance, current routine laboratory testing is relevant in day-to-day care; patients with new CMV infection and even patients who have undergone CMV infection might profit from further individualized risk stratification.

## 5. Conclusions

CMV infection before and after LT might contribute to the pathogenesis of CKD in the LT population and therefore play a pivotal role in contributing to major comorbidities that are relevant in the setting of aftercare. However, the pathophysiological mechanisms remain unclear; their identification requires further clinical and experimental research.

## Figures and Tables

**Table 1:** Patient cohort’s characteristics.

	Patient Cohort (*n* = 745)
Median age at LT in years (min–max)	56.3 (19.3–74.2)
Sex (%) Male Female	494 (66.3) 251 (33.7)
Indication for LT (%) ALD Viral hepatitis AIH/PSC/PBC NASH/cryptogenic cirrhosis HCC/CCA ALF Others	304 (40.8) 168 (22.6) 97 (13.0) 58 (7.8) 32 (4.3) 25 (3.4) 61 (8.2)
Median MELD (min–max)	15 (6–40)
Comorbidities at LT (%) Cardiovascular disease Diabetes	79 (10.6) 65 (8.7)
Median eGFR at LT mL/min (min–max)	84.8 (5–150)
CKD at LT (%) I II III IV V	346 (46.4) 237 (31.8) 118 (15.8) 28 (3.8) 16 (2.1)
Donor-recipient CMV risk stratification (%) R negative/D negative R negative/D positive R positive/D negative R positive/D positive Missing data	82 (11.0) 146 (19.6) 174 (23.4) 257 (34.5) 86 (11.5)
CMV infections after LT (%) Time from LT <90 days >90 days Median viral load in IU/mL (min–max) At diagnosis Peak Manifestation (%) CMV viremia CMV syndrome CMV tissue invasion Treatment (%) Observation only IS reduction (val)ganciclovir	271 (36.4) 220 (81.2) 51 (18.8) 3160 (10–13,900,000) 7335 (756–13,900,000) 203 (27.2) 66 (8.9) 2 (0.003) 61 (22.5) 34 (12.5) 176 (64.9)
Maintenance IS after LT (%) CNI Tacrolimus Cyclosporin A MMF mTORI Combination therapy	725 (97.3) 625 (83.8) 100 (13.4) 353 (47.4) 81 (10.9) 476 (63.9)
Mean time of follow-up in months (SD)	117.2 (58.3)
Patients deceased (%) Cause of death Graft failure Neoplasms Cardiovascular Pulmonary Infection Neurological Acute bleeding Trauma Other	313 (42.0) 38 (5.1) 108 (14.5) 36 (4.8) 9 (1.2) 68 (9.2) 7 (0.9) 10 (1.3) 5 (0.7) 32 (4.3)

LT—liver transplantation; ALD—alcoholic liver disease; AIH—autoimmune hepatitis; PSC—primary sclerosing cholangitis; PBC—primary biliary cirrhosis; NASH—non-alcoholic steatohepatitis; HCC—hepatocellular carcinoma; CCA—cholangiocellular carcinoma; ALF—acute liver failure; eGFR—estimated glomerular filtration rate; CKD—chronic kidney disease; CMV—cytomegalovirus; CNI—calcineurin inhibitor; MMF—mycophenolate mofetil; mTORI—mammalian target of rapamycin inhibitor.

**Table 2:** Impact of clinical parameters on eGFR and CKD prior to LT in univariate analysis. Clinical variables with putative impact on renal function using either the CKD classification or eGFR were explored.

Variable	Groups	CKD I *n* (%)	CKD II *n* (%)	CKD III *n* (%)	CKD IV *n* (%)	CKD V *n* (%)	*p*	eGFR Prior to LT in mL/min Median (Min–Max) *	*p*
Age	≤60 years >60 years	254 (50.3) 92 (38.0)	147 (29.1) 90 (38.0)	72 (14.3) 46 (19.2)	21 (4.2) 7 (2.9)	11 (2.2) 5 (2.1)	0.019	90.0 (8–150) 81.4 (11–131)	*p* = 0.011
Sex	Male Female	256 (51.8) 90 (35.9)	155 (31.4) 82 (32.7)	67 (13.6) 51 (20.3)	13 (2.6) 15 (6.0)	3 (0.6) 13 (5.2)	<0.001	90.0 (10–150) 71.4 (5–137)	<0.001
Indication for LT	ALD Viral hepatitis AIH/PSC/PBC NASH/cryptogenic cirrhosis HCC/CCA ALF Others	128 (42.1) 74 (44.0) 55 (56.7) 24 (41.1) 20 (62.5) 13 (52.0) 32 (52.5)	106 (34.9) 60 (35.7) 29 (29.9) 16 (27.6) 8 (25.0) 4 (16.0) 14 (23.0)	57 (18.8) 25 (14.9) 9 (9.3) 12 (20.7) 2 (6.3) 3 (12.0) 10 (16.4)	9 (3.0) 6 (3.6) 1 (1.0) 3 (4.9) 1 (3.1) 5 (20.0) 3 (4.9)	4 (1.3) 3 (1.8) 3 (5.2) 3 (5.2) 1 (3.1) 0 (0) 2(3.3)	0.005	82.7 (10–123) 83.0 (12–131) 90.0 (11–133) 69.5 (8–137) 90.5 (12–135) 90.0 (17–150) 90.0 (5–147)	0.038
Cardiovascular disease	-	23 (29.1)	30 (38.0)	20 (25.3)	5 (6.3)	1 (1.3)	0.008	62.0 (11–118) 88.1 (5–150)	<0.001
Diabetes	-	29 (44.6)	23 (35.4)	13 (20.0)	0 (0.0)	0 (0.0)	0.256	78.4 (30–130) 85.4 (5–150)	0.82
Recipient CMV status	CMV positive CMV negative	209 (43.8) 132 (52.0)	153 (32.1) 77 (30.3)	82 (17.2) 35 (13.8)	24 (5.0) 4 (1.6)	9 (2.4) 6 (1.9)	0.059	81.4 (8–137) 90.0 (5–147)	0.004
Median MELD	-	14 (6–40)	14 (6–40)	20 (6–40)	32 (15–40)	36 (20–40)	<0.001	r = −0.323 **	<0.001

LT—liver transplantation; ALD—alcoholic liver disease; AIH—autoimmune hepatitis; PSC—primary sclerosing cholangitis; PBC—primary biliary cirrhosis; NASH—non-alcoholic steatohepatitis; HCC—hepatocellular carcinoma; CCA—cholangiocellular carcinoma; ALF—acute liver failure; CMV—cytomegalovirus; MELD—model of end-stage liver disease. * median and range only for categorical variables. ** Pearson correlation coefficient was calculated for correlation of metric MELD and eGFR).

**Table 3:** Multivariate analysis of impact of variables on eGRF prior to LT.

Variable	*p*	Regression Coefficient	95% CI	
			Lower	Upper
Age (ref.: ≤60 years)	<0.001	−7.29	−11.42	−3.17
Sex (ref.: male)	<0.001	−12.52	−16.70	−8.34
Indication for LT	0.015	1.49	0.29	2.70
Cardiovascular disease (ref.: yes)	0.004	8.90	2.84	14.96
Recipient CMV status (ref.: CMV negative)	0.117	−3.22	−7.25	0.81
MELD	<0.001	−0.88	−1.07	−0.69

LT—liver transplantation; CMV—cytomegalovirus.

## Data Availability

The data that support the findings of this study are available on request from the corresponding author. The data are not publicly available due to privacy and ethical restrictions and the local institution’s data policy.

## Supplementary Materials

The following supporting information can be downloaded at: https://www.mdpi.com/article/10.3390/v16121830/s1, Table S1: Course of eGFR after LT, dependent on CMV infection.
