# Supplementary material for: The Association Between Cytomegalovirus Infection and Kidney Damage in the Liver Transplant Setting
Source: Viruses. 2024 Nov 26;16(12):1830. doi: 10.3390/v16121830 (PMC11680441; doi:10.3390/v16121830)
Supplement: Supplementary file 1 [file viruses-16-01830-s001.zip › viruses-3264718-supplementary.pdf]

	eGFR prior to LT	eGFR 6 months after LT	eGFR 12 months after LT	eGFR 36 months after LT	eGFR 60 months after LT	eGFR 120 months after LT	eGFR 180 months after LT
**Group 1: no CMV pre/post LT (min-max); [n]**	90.0 (5-150);[186]	89.3 (22-130); [182] 0.7 (0.75)	83.4 (14-130); [182] 5.9 (0.15)	81.6 (14-146); [148] 1.8 (0.63)	80.8 (36-122); [112] 0.8 (0.22)	63.5 (15-110); [58]	67.0 (48-99); [8]
Loss of eGFR to prior (*p*)	-	0.7 (0.75)	6.6 (0.105)	8.4 (0.05)	9.2 (0.003)	17.3 (<0.001)	-3.5 (0.40)
Total loss of eGFR (*p*)	-					26.5 (<0.001)	23.0 (0.09)
**Group 2: CMV+R (min-max); [n]**	84.3 (8-137);[288]	86.1 (8-127); [281]	84.1 (16-128); [278]	82.9 (14-123); [235]	79.4 (26-122); [207]	63.0 (5-116); [106]	72.0 (37-105); [12]
Loss of eGFR to prior (*p*)	-	-1.8 (0.26)	2.0 (0.71)	1.2 (0.16)	3.5 (0.41)	16.4 (<0.001)	-9.0 (0.46)
Total loss of eGFR (*p*)	-	-1.8 (0.26)	0.2 (0.42)	1.4 (0.62)	4.9 (0.20)	21.3 (<0.001)	12.3 (0.23)
**Group 3: CMV-infection post-LT (min-max); [n]**	79.9 (10-137);[271]	83.3 (11-139); [263] -3.4 (0.55)	80.7 (12-135); [256]	75.0 (6-120); [205]	72.7 (8-121); [167]	57.0 (8-118); [61]	36.0 (21-82); [5]
Loss of eGFR to prior (*p*)	-	-3.4 (0.55)	2.6 (0.96)	5.7 (0.07)	2.3 (0.19)	15.7 (<0.001)	21.0 (0.28)
Total loss of eGFR (*p*)	-		-0.8 (0.57)	4.9 (0.27)	7.2 (0.02)	22.9 (<0.001)	43.9 (0.05)
**Overall (min-max); [n]**	84.8 (5-150); [745]	86.2 (8-139); [726]	82.8 (12-135); [716] 3.4 (0.36)	79.0 (6-146); [588]	77.6 (8-121); [486]	61.0 (5-118); [225]	61.0 (21-105); [25]
Loss of eGFR to prior (*p*)	-	-1.4 (0.37)	2.0 (0.94)	3.8 (0.30)	1.4 (0.061)	16.6 (<0.001)	0 (0.58)
Total loss of eGFR (*p*)	-	-1.4 (0.37)		5.8 (0.061)	7.2 (<0.001)	23.8 (<0.001)	23.8 (0.01)
p (group 1, 2, 3)	0.004	0.006	0.071	0.011	0.005	0.114	0.106

CMV – cytomegalovirus; n – number; median eGFR, unit: ml/min

Supplement Table S1 Course of eGFR after LT dependent of CMV-infection
